# College and Hospital Notes

**Published:** 1910-04

**Authors:** 


### COLLEGE AND HOSPITAL NOTES.

---

THE following extension course of lectures, Tulane Medical Department of New Orleans, will be delivered on the days, dates and hours mentioned, at the Hutchinson Memorial, corner Canal and Villere Streets. The general public, the medical profession and all medical students are invited to attend these lectures according to schedule.

Lectures which are purely professional, in which the general public would not be interested, are indicated (*).

*Wednesday, April 6, 8 P. M., Professor Charles Chassaignac, Sexual Continence and Prophylaxis. This lecture is for the medical profession, students and male public only.

*Tuesday, April 12, 8 P. M., Professor Paul Michinard, The Management of Labor. This lecture is for the medical profession and students only.

Wednesday, April 13, 8 P. M., Professor J. A. Storck, Hygiene of the Gastrointestinal Tract. Medical profession, students, and general public invited.

*Tuesday, April 19, 8 P. M., Professor C. J. Miller, The Anatomical Basis Underlying Injuries and Repair of The Pelvic Floor. This lecture is for the medical profession and students only.

Wednesday, April 20, 8 P. M., Professor Irving Hardesty, The Anatomy of the Ear in Relation to the Theories of Hearing. Medical profession, students and general public invited.

Wednesday, April 27, 8 P. M., Professor J. T. Halsey, Physiological Action of Some Much Used Drugs with Demonstrations. Medical profession, students and general public invited.

The lectures for March are omitted.

Alumni week, will be May 9-15, 1910.

---

The Buffalo General Hospital Alumni Association held its annual meeting and dinner Thursday evening, March 10, 1910, in the Lafayette Hotel. After singing all the songs on the calendar, the doctors listened to some talks on various phases of the profession and as an aftermath enjoyed a comedy by themselves, as well as a few sketches of themselves and then cleared the boards for the Surgical Millenium in Buffalo, "a tragediette in one-fourth of an act." Dr. E. L. Shurley, of Detroit, was toastmaster. The speakers were Drs. Charles Stockton, H. U. Williams, Roswell Park, Marshall Clinton and H. A. Maynard.

---

A reunion of the medical graduates of McGill University, Montreal, will be held June 6 and 7, 1910. The new buildings which are being erected for the accommodation of the medical faculty, replacing those which were destroyed by fire three years ago, are now approaching completion and will be ready for occupation in the early summer. The medical faculty has therefore decided, with the sanction and approval of the principal and the governors of the university, to hold the next annual convocation, for the conferring of degrees in medicine in the new building and to arrange for the formal opening ceremonies at the same time; and to further signalise the event by carrying out a long contemplated plan for a reunion of all her graduates.

His Excellency, the Governor-General, has consented to be present, and a provisional program has been arranged. All graduates are cordially invited to be present.

---

The Buffalo Children's Hospital is about to establish a new home for nurses and a new pavilion for contagious diseases through the generosity of Mrs. William Hamlin and other friends of the institution. It will be remembered that Mrs. Charles W. Pardee built the new hospital.

Mrs. Hamlin has written the managers of the hospital that she has bought the property adjoining the hospital on the west and will present it to the board of managers, with the distinct stipulation that money is raised for the new home. This, properly equipped, will cost about $45,000. The new contagious pavilion will cost about $30,000, of which $12,000 has already been raised, while Mr. Williams Lansing, the architect, has drawn the plans and given his services free, in memory of his little son.

To enable the managers to take advantage of Mrs. Hamlin's offer of the site they now appeal to the people of Buffalo for funds. Contributions may be sent to Mr. J. N. Adam, No. 60 Oakland Place, chairman of the advisory board, or to Mrs. Lester Wheeler, No. 623 Delaware Avenue, president of the board of managers.

